# Effect of Integrative Chinese and Western Medicine Therapy on Long-Term Clinical Outcomes in Patients with Heart Failure: A Real-World Study Including 394 Patients

**DOI:** 10.1155/2022/2001397

**Published:** 2022-09-13

**Authors:** Zihan Wang, Jin Zhang, Gaoyu Zhang, Tianyi Lan, Ziyi Sun, Xiaoyan Lu, Li Huang, Lin Li

**Affiliations:** ^1^Beijing University of Chinese Medicine, Beijing, China; ^2^Department of Integrative Cardiology, China-Japan Friendship Hospital, Beijing, China

## Abstract

**Objective:**

To explore the effect of integrative Chinese and Western medicine therapy on the clinical outcomes of patients with heart failure.

**Methods:**

This is a retrospective cohort study in the real world. Patients were divided into “conventional therapy” and “integrative therapy” groups according to treatment modality. The occurrence of cardiovascular events (CVE) was determined during follow-up. Survival curves were plotted, and survival analysis was performed using Cox regression to report survival in both groups. Further subgroup tests were performed as sensitivity analyses. A Markov model was constructed to predict patients with distant heart failure conditions based on real follow-up data.

**Results:**

Based on diagnostic criteria, 394 patients with heart failure were included. The integrative therapy group had (*N* = 181) older patients (*P*=0.005), higher proportion of renal insufficiency (*P* < 0.001), higher creatinine (*P*=0.040), hypersensitive C-reactive protein (*P*=0.007), N-terminal pro-B type natriuretic peptide (*P*=0.019) levels, more patients in cardiac function class IV (*P*=0.004), and longer hospital days (*P*=0.003) than the conventional therapy group (*N* = 213). Survival was better in the integrative therapy group than in the conventional therapy group (log-rank *P* < 0.001). Multifactorial Cox regression identified “conventional therapy” or “integrative therapy” as an independent factor affecting the risk of CVE in patients with heart failure, with the risk of CVE being lower in the integrative therapy group (HR = 0.322, 95% CI = 0.185–0.561). A subgroup analysis found no significant association between therapy modality and risk of CVE in older patients (age ≥65 years, *P*=0.210) and those who had renal insufficiency (*P*=0.062). The Markov model predicted better cardiac function in the integrative therapy group than in the conventional therapy group at all time points (all *P* < 0.001).

**Conclusion:**

In patients with heart failure, integrative therapy of Chinese and Western medicine had better long-term outcomes than conventional therapy. However, patients with advanced age and renal insufficiency had no significant advantage. *Trials Registration*. This trial is registered with China Clinical Trials Registry, ChiCTR2100050927, registered 8 September 2021, https://www.chictr.org.cn/showproj.aspx?proj=133451.

## 1. Introduction

Heart failure (HF) is a clinical syndrome involving a progression of cardiovascular diseases to severe stages, which are associated with critical illness, high fatality, and complex therapy [1]. Traditional therapy regimens for heart failure, with angiotensin-converting enzyme inhibitors (ACEI) or angiotensin-receptor blockers (ARB), beta-blockers, and mineralocorticoid receptor antagonist (MRA) as mainstay [2], have clear benefits but still fail to meet the therapeutic needs of an increasing number of patients. As patients vary, individualised and specific medication regimens need to be promoted for specific disease management to improve benefits to patients [3]. Integrative therapy in Chinese and Western medicine is one of the important individualised therapy modalities. Individualised integrative therapy of Chinese and Western medicine can improve the clinical symptoms of patients with heart failure with satisfactory results compared to single standardised Western medicine treatment [4–6]. However, the effect of integrative Chinese and Western medicine therapy on the long-term clinical outcome of patients with heart failure has not been widely reported clinically, and the effect on clinical progression, readmission rates, and mortality is still not well understood.

The Markov model [7] is a decision analysis method that can model disease trends and prognosis and determine the efficacy and economic advantages of therapy [8]. It is increasingly being used in medical research [9, 10] to predict patient disease progression and save research costs. In this study, patients with heart failure were followed up to observe the occurrence of cardiovascular events (CVE) by describing their disease progression using survival analysis and then using Markov models for decision analysis to simulate long-term disease trends. The study sought to elucidate the effect of integrative Chinese and Western therapy on the long-term clinical outcomes of patients with heart failure in a real-world context and provide new diagnostic ideas and evidence-based evidence for the precise prevention and control of heart failure.

## 2. Information and Methods

### 2.1. Selection Method

A retrospective information search was performed through the medical record system of the China-Japan Friendship Hospital. From July 2016 to July 2021, all patients with heart failure admitted to the Department of Integrative Cardiology at China-Japan Friendship Hospital were screened. Patients aged 18–75 years who had their first hospital admissions for heart failure and could complete follow-up were included. Patients with severe liver and renal abnormalities (transaminases >3-fold elevated or glomerular filtration rate <15 ml·min^−1^·1.73 m^−2^), those with severe haematopoietic and endocrine system abnormalities, and those whose data collection was hampered by factors, such as mental language, were excluded. Additionally, patients using intravenous preparations of traditional Chinese medicine and proprietary Chinese medicines and patients using new antiheart failure drugs, such as sacubitril/valsartan and SGLT2 inhibitors, were excluded.

The study was approved by the Clinical Research Ethics Committee of China-Japan Friendship Hospital (2019-120-K82), conducted in accordance with the Declaration of Helsinki, and registered with the China Clinical Trials Registry (ChiCTR2100050927). All patients provided written informed consent. The specific patient inclusion process is shown in supplementary materials (Figure S1).

### 2.2. Diagnostic Criteria

The diagnostic criteria for heart failure were based on the ESC Guidelines [2]. Overall, patients had to have significant symptoms or signs with abnormalities in left ventricular ejection fraction (LVEF), left heart structural function, and biomarkers of heart failure to be diagnosed with heart failure.

### 2.3. Research Methods

#### 2.3.1. Basic Information

General information about the study population, including demographic information (gender, age, height, and weight), underlying disease history (coronary heart disease, hypertension, heart valve disease, diabetes mellitus, arrhythmia, and renal insufficiency), and the main symptoms of the patients, was recorded and assessed for New York Heart Function Classification [11] (NYHA classes Ι–IV).

#### 2.3.2. Index Testing

The venous blood specimens of all patients were collected during a fasting state in the early morning of the second day after admission and tested for haemoglobin (Hb), hypersensitive C-reactive protein (Hs-CRP), low-density lipoprotein cholesterol (LDL-C), and N-terminal pro-B type natriuretic peptide (NT-proBNP) at the Laboratory Department of the China-Japan Friendship Hospital. An independent sonographer performed echocardiography, measuring and calculating common indices, including 2D ultrasound, recording LVEF, left ventricular end-diastolic diameter (LVIDd), left ventricular posterior wall thickness (PWTd), septal thickness (SWTd), and left ventricular mass index (LVMI), with the following algorithm.

1. Based on the DEVEREUX-corrected left ventricular mass (LVM) calculation formula [12]: LVM (g) = 0.8 × {1.04 [(LVIDd + PWTd + SWTd)^3^ − LVIDd^3^]} + 0.6.
2. Body surface area (BSA) formula: BSA (m^2^) = 0.0061 × height (cm) + 0.0128 × weight (kg) − 0.1529.
3. Final calculation of LVMI (g·m^−2^) = LVM/BSA.

#### 2.3.3. Therapy Modalities

All patients received the standardised Western medicine therapy, including the use of diuretics, ACEI/ARB, *β*-blockers, and MRA, recommended by the ESC Guidelines [2]. On this basis, some patients received oral doses of Chinese herbal tonics according to their own wishes or doctor's recommendations. The attending physician assessed the condition and identified the symptoms, prescribed individualised Chinese herbal medicine for oral consumption, and retrieved the information on the medical prescriptions contained in the medical record management system. Patients who continued to take Chinese medicine for >6 months per year were included in “integrative therapy,” and those who received only standardised Western medicine therapy were included in “conventional therapy.” All patients were maintained on a continuous and regular basis, with specific medication adjusted at follow-up appointments, with 6 months being the treatment cycle.

#### 2.3.4. Follow-Up and Outcome

Patients were followed up retrospectively in person or by telephone at 6-month intervals to assess changes in cardiac function classification after discharge and to record the occurrence of CVE (including readmission due to heart failure, acute coronary syndrome, stroke, and death from cardiovascular causes). The occurrence of “CVE” was the primary outcome. The minimum follow-up period for each patient was 6 months, and the maximum follow-up period was 36 months. The total follow-up period ranged from 6 months to 36 months, depending on the time of inclusion.

#### 2.3.5. Sample Size Calculation

Preestimating the 5–10 variables that may be associated with the occurrence of CVE in patients with heart failure, and a minimum sample size of approximately 50–100 patients with CVE was required to follow the principle of approximately 10 outcome events per variable in regression analysis [13]. The final study cohort comprised 394 patients with heart failure.

### 2.4. Algorithm of Markov Model

#### 2.4.1. Markov Model Setting

Based on the conventional and integrative therapy groups, five model states (cardiac function classes Ι–IV and CVE) were set according to the evolution of patients with heart failure and principles of Markov model construction [7]. The model states were used to simulate the disease progression process, which could either remain unchanged in one state or be transferred to other states. The patient's cardiac function class at the time of inclusion in the study was the starting state. At each cycle, the model state could be transferred to other cardiac function classes. At the occurrence of a CVE, it was transferred to the absorbing state (which could not be transferred to other states). The logic was as follows: (1) Patients with heart failure received conventional or integrative therapy, respectively, and all patients might experience the five model states described above. (2) Patients with NYHA Ι–IV could be classified as event-free or having CVE occurrence after treatment cycles. (3) Patients were classified as event-free at each cycle transition to NYHA classes I–IV and then continued to the next cycle, whereas patients with CVE had their cycles terminated.

Patients were observed for cardiac function from baseline to 6 months of treatment to determine the frequency of occurrence of the five model states, which was used to calculate the model probability of state shift. Patients were included for a maximum of 36 months of actual observation, a model cycle of 6 months was set, and a cycle time of 60 months was used to finally calculate the cumulative probability of each Markov state.

#### 2.4.2. Markov Model Evaluation

Markov models can predict changes in disease over time based on the current state and probability [14]. It is essentially a statistical predictive model that requires model evaluation to verify veracity. The chi-square goodness-of-fit test was used for model evaluation to test the distribution of cardiac function in the sample and compare the degree of agreement between predicted and actual frequencies.

### 2.5. Statistical Processing

Statistical analysis and visualization were performed with R language (V4.1.1, AT&T Bell Laboratories). Normally distributed data were expressed as mean ± standard deviation, nonnormally distributed data were expressed as median and quartiles, and count data were expressed as percentages. In comparing the sample means of two groups, the independent samples *t*-test was used for normally distributed data, the rank sum (Kruskal–Wallis *H*) test was used for nonnormally distributed data, and the chi-square test was used for count data.

In the survival analysis, the main observation was whether the different treatment modalities had an impact on the survival outcome of patients with heart failure. Survival curves were first plotted with Kaplan–Meier curves, and the differences between survival curves were compared using the log-rank test. Multifactorial Cox regression was then used to evaluate whether the different treatment modalities realistically affected the survival process. Cox regression models were evaluated using C-Statistics and time-dependent receiver operating characteristic curves (ROC). Finally, sensitivity analyses of the study results were performed to test for corrected associations between the target variables and survival outcomes by dividing the subgroups and stratifying them.

The Markov model was constructed and computationally analysed with the decision software TreeAge Pro 2011 (Software, Inc.). The data obtained from the follow-up visits at each time point were included in the constructed model to calculate the respective probabilities of transfer of cardiac function status. Further state prediction of the heart failure disease progression process was performed, and the cumulative probability of cardiac function state at each time point was calculated cyclically.

## 3. Results

### 3.1. Clinical Characteristics of Patients with Heart Failure

A total of 394 patients who met the diagnostic criteria for heart failure were included in the study; they included 181 patients in the “integrative therapy” group and 213 patients in the “conventional therapy” group. The basic information of the two groups of patients is shown in Table 1, and the overall population information is shown in supplementary materials (Table S1).

No significant differences were found in the overall gender, weight, body mass index (BMI), medication use, underlying disease history (hypertension, coronary artery disease, arrhythmia, heart valve disease, and diabetes mellitus), smoking history, laboratory tests (including Hb, TG, LDL-C, Hcy, K^+^, Na^+^, Cl^−^, and Mg^2+^), and echocardiographic indices, LVEF and calculated LVMI, between the two groups (*P* > 0.05). The integrative therapy group had relatively older patients (*P*=0.005), higher proportion of combined renal insufficiency (*P* < 0.001), higher test Cr level (*P*=0.04), higher Hs-CRP level (*P*=0.007), higher NT-proBNP (*P*=0.019) level, more patients with poorer heart function (cardiac function class IV) (*P*=0.004), and relatively longer hospital stays than the conventional therapy group (*P*=0.003).

The results reflect the real situation in clinical practice. When clinicians see patients who are elderly or in poor general condition, they are more likely to prescribe individualised integrative therapy to improve their condition more comprehensively. Additionally, correspondingly longer consultation times are required.

### 3.2. Survival Analysis

#### 3.2.1. Patient Survival and Kaplan–Meier Survival Analysis

**Figure 1:**
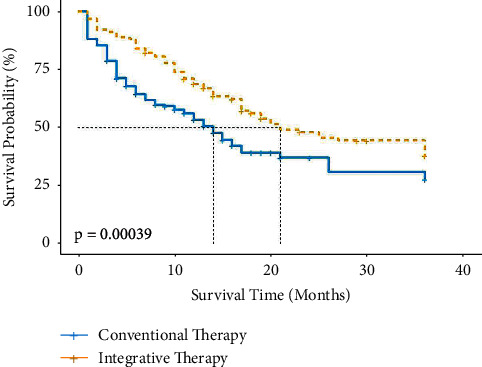
Survival curves based on the Kaplan–Meier method for two groups of patients.

Patient survival was observed, and the survival course of patients in the integrative therapy group was compared with that of the conventional therapy group. Survival times are shown in Table 2.  Figure 1 demonstrates the survival curves based on these data. The survival curves of patients in the integrative therapy group were consistently higher than those of patients in the conventional therapy group. The difference in survival curves between the two groups was tested using the log-rank test (log-rank *P* < 0.001), which indicated that patients in the integrative therapy group survived better than those in the conventional therapy group.

#### 3.2.2. Multivariable-Adjusted Cox Regression Analysis

We further used multivariable-adjusted multifactorial Cox regression to assess the difference between the effects of the two therapy modalities on the survival course of patients with heart failure.

To streamline the data variables, we transformed some of the continuous variables into categorical variables, as described in supplementary materials (Table S2). LDL-C, Hcy, Hs-CRP, Na^+^, K^+^, Mg^2+^, Cl^−^, in-hospital cardiac function class, NT-proBNP, LVEF, and LVMI were used as covariates to construct a multimodal multifactorial Cox regression model. During the construction of the multimodal model, model Ι was corrected for age and gender; model II was adjusted for BMI, combined medications, underlying disease history, smoking history, comorbidities, and in-hospital basal cardiac function class; model III was further corrected for continuous variables, such as laboratory test results, LVEF, and LVMI. Model III was used as the main study model.

The proportion of patients in the integrative therapy group who developed CVE was similar to that in the conventional treatment group (*P*=0.545). The risk ratio between treatment modality and the development of CVE, with 95% confidence intervals, was demonstrated by Cox regression analysis, as shown in Table 3. Therapy modality, whether “integrative” or “conventional,” was found to be an independent factor influencing the development of CVE in patients with heart failure (*P* < 0.001). Patients with heart failure receiving combined Western and Chinese treatment had a lower risk of developing CVE than patients in the conventional therapy group (HR = 0.322, 95% CI = 0.185–0.561, *P* < 0.001). Additionally, patients with combined coronary artery disease (HR = 2.334, 95% CI = 1.385–3.930, *P*=0.001) were at higher risk of CVE.

#### 3.2.3. Model Evaluation

**Figure 2:**
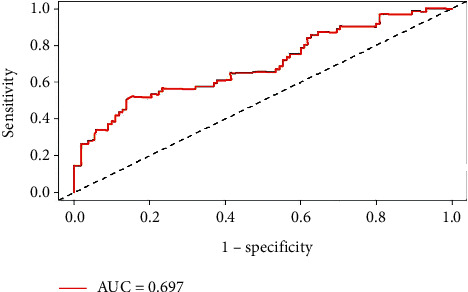
ROC curves at “1-year” follow-up.

The Cox regression model (model III) was statistically evaluated with a negative 2-fold log-likelihood value of 626.090 and an Omnibus test, *χ*^2^, of 29.049 (*P* < 0.001), indicating that the overall model test was statistically significant. A test of model discrimination was performed to obtain C-Statistics, calculated as C-Statistics = 0.701. Additionally, we evaluated the model using a time-dependent ROC curve, as shown in Figure 2, with the risk rate of CVE at 12 months (1 year) of follow-up as the outcome (area under the ROC curve (AUC) = 0.697). These results all demonstrate that the multifactor Cox regression model (model III) constructed in this study was highly accurate and representative of the cohort population.

#### 3.2.4. Subgroup Analysis

The baseline clinical data revealed differences in age, the proportion of patients with combined renal insufficiency, and basal (in-hospital) cardiac function class between the two groups. In conjunction with the additional results in Section 3.2.2 of the study (patients with combined coronary artery disease were at higher risk of CVE), further subgroup tests for sensitivity analysis of the study results were performed. All patients were stratified according to age (overall median age of 65 years), basal heart function class (class II, III, or IV), combined renal insufficiency (yes or no), and coronary artery disease (yes or no). The results of the subgroup analysis based on the Cox regression model III tests are presented in Table 4.

Upon examining the group of patients who were relatively older (age ≥65 years, *P*=0.210) and had coexisting renal insufficiency (*P*=0.062), we found that no statistically significant correlation was observed between the different treatment modalities and the occurrence of CVE, suggesting that, in patients with more severe disease (including but probably not limited to older age and poorer renal function), integrative therapy does not necessarily result in greater benefit. No statistically significant effect was found in patients with class II cardiac function due to the small sample size (*N* = 46).

With the exception of these findings, the final results did not change substantially in most subgroup analyses. The correlation between treatment modality and CVE occurrence was consistent with that of the main model (model III) after adjusting for age, cardiac function class, and history of renal insufficiency and coronary artery disease.

### 3.3. Markov Model-Based Efficacy Evaluation

#### 3.3.1. Frequency of Status Change and Frequency Matrix

In the next study, we used the patient's cardiac function classification at discharge as the base value (two patients in the integrative therapy group died during hospitalization, compared to admission). Patients' cardiac function classification 6 months after treatment was recorded. The changes in cardiac function status from discharge to the sixth month in both groups are shown in supplementary materials (Tables S3 and S4). These changes demonstrate the frequency of transfer from the original cardiac function status to the new status.

#### 3.3.2. Prediction of Cardiac Function States

The transfer frequencies recorded were used as model transfer probabilities to predict the overall distribution of cardiac function over 5 years for patients with heart failure in the conventional and integrative therapy groups using Markov models.

Table 5 demonstrates the predicted overall frequencies of cardiac functional status over 5 years for both groups of patients. The proportion of patients with heart function classes III-IV was significantly reduced in both groups after long-term standardised medication, compared to the initial period of treatment, with more patients improving and stabilising in heart function classes I-II. Due to the progression of heart failure and natural physiological aging, the incidence of CVE in both groups inevitably increased to some extent, but the incidence of CVE in the conventional therapy group was higher than that in the integrative therapy group. Additionally, the overall cardiac function in the integrative therapy group was better than that in the conventional therapy group at different time periods (all *P* < 0.001).

#### 3.3.3. Model Evaluation

The actual frequency of cardiac function classification in the different treatment groups after 1 year of follow-up was compared with the predicted probability of cardiac function classification obtained by running the Markov model (in Table 6) and evaluated using the chi-square goodness-of-fit test. No statistically significant differences existed between the predicted and actual frequencies of cardiac function in both the conventional therapy group (*P*=0.200) and the integrative therapy group (*P*=0.089). The model fit was good, and the Markov model could be better applied to this study cohort.

## 4. Discussion

The overall probability of survival in patients with heart failure rapidly declined during the first 6 months. This decline was probably due to the “acute vulnerable period” [15] after their first hospitalization for heart failure, during which period patients do not have a stable cardiac function. This suggests that clinicians need to improve the postdischarge management of patients with initial CVE to reduce the risk of recurrent CVE. Concurrently, the probability of survival was at a stable, almost unchanging plateau after approximately 24 months of follow-up, which was probably related to the number of patients included and the insufficient number of patients in “survival” status. Unequivocally, the survival curves suggest a lower risk of CVE in the integrative therapy group, which was confirmed in a subsequent multivariate corrected Cox regression analysis. Therefore, compared to conventional therapy, integrative therapy could reduce the risk of CVE by approximately 67.8% in patients with heart failure (HR = 0.322, 95% CI = 0.185–0.561, *P* < 0.001).

Additionally, the Cox regression analysis revealed that the occurrence of CVE in the patient population was associated with the presence of coexisting coronary artery disease. Coronary heart disease is one of the clear primary causes of heart failure [16]. It plays an important predisposing and contributing role in the development of heart failure. Our results are consistent with the findings of earlier studies and suggest that clinicians should pay much attention to the early treatment of patients with heart failure with underlying coronary heart disease.

Unfortunately, regardless of the treatment modality, half of the outcome events in the overall population of patients with heart failure occurred before 17 months (median survival time), which is somewhat representative of the prognosis of the population of patients with heart failure in Beijing, China [17, 18]. The use of individualised combination therapy, if promoted, may improve the long-term clinical outcome of patients.

Further subgroup analysis showed some interesting variations. In the group of patients who were older (defined as ≥65 years in this study) or had coexisting renal insufficiency, integrative therapy did not significantly improve patient prognosis compared with conventional therapy. This finding is not consistent with the experience of clinicians specialising in traditional Chinese medicine, who usually prefer integrative therapy with Chinese medicine for older patients with multiple comorbidities and believe that this approach results in greater benefit to patients [19]. Notably, the management of elderly patients and those with coexisting renal insufficiency is often more complex; they may be less responsive and sensitive to therapeutic agents [20, 21]. Therefore, individualised integrative therapy for heart failure only may no longer be effective in improving the condition. More varied integrative therapies, such as health education, rehabilitation exercises, and risk stratification management [22], may be of interest to achieve better outcomes.

Due to the limited number of cases and the difficulty of follow-up, our study only observed changes over a 3-year period, which was not sufficient to demonstrate the benefits of integrative therapy in improving long-term clinical outcomes in patients with heart failure. Therefore, we introduced a predictive tool for decision analysis, the Markov model, to assist in this study. Thereafter, we simulated the progression of patients over a 5-year period.

The Markov model is one of the most commonly used decision analysis methods [23]. It differs from traditional modelling based on methods, such as Cox regression, in that it focuses on the transfer between states and lag time and can analyse the influence of risk and regression factors in the disease process. It has the advantage of modelling the development of chronic complex diseases [24] and can be used for disease assessment of cardiovascular diseases [25]. In this study, we applied the Markov model to the field of heart failure disease.

Markov model, as a method of data analysis, requires the construction of models based on the actual evolution of patients with heart failure as a means of predicting changes in disease over many years, which cannot be done without accurate data observed in the real world. In calculating the probability of transfer of heart function status within the model, we selected the change in heart function classification from baseline (when the patient was discharged from the hospital) to 6 months of follow-up as the source of data. The number of valid samples within this time frame is sufficient, and the changes in patients' conditions are representative. Therefore, the probability of transfer can be guaranteed to be relatively accurate. Furthermore, the chi-square test showed that the Markov model-predicted cardiac functional class at this probability of transfer was consistent with the actual follow-up results, suggesting that the model probabilities and the long-term changes in condition predicted by the model were reliable.

The results of the Markov model loop showed that the overall functional status of patients with heart failure failed to improve significantly over time, even with pharmacological interventions. Most patients experienced CVE at various times, and only a few patients remained asymptomatic (NYHA class I). From the survival status of patients with heart failure at the 1, 3, and 5 time points after model fitting, patients in the integrative therapy group were in a better position overall, with relatively slower deterioration in cardiac function and a lower incidence of CVE. Notably, in calculating the Markov model probabilities, the actual CVE percentages for the two groups of patients at the 1-year (12-month) time point were 45.2% and 52.5%, respectively, whereas the cumulative CVE risk probabilities for patients at 12 months observed in the survival analysis with the Kaplan–Meier method (Figure 1) were around 30% and 45%, which were due to the differences in the calculation method. The Markov model requires calculations based on those currently in the cohort (the denominator already excludes patients with dislodgement and prior CVE), whereas the Kaplan–Meier method requires consideration of additional censored cases in the calculations (the denominator does not exclude patients with dislodgement and prior CVE). Therefore, the proportion obtained from the Markov model calculation was larger.

This study had a few limitations. First, study participants were recruited from the same hospital and almost the same city (Beijing). The single-centre nature may have introduced bias in the collection of clinical data. Second, of greater concern is that the risk of CVE in patients with heart failure should increase over time. However, in applying the Markov model, the risk rate was treated as a fixed value and calculated as the probability of transfer between 0 and 6 months, which should be dynamic in the real world. This was not possible in this study due to the small number of valid cases at the end of the follow-up period and the lack of actual data available. In future real-world studies, the number of patients in the cohort could be expanded to construct a more accurate “time-dependent” Markov model [26], in which the probability of transfer varies over time to give a more realistic picture of the disease progression.

## 5. Conclusion

In this study, integrative therapy or conventional therapy was an independent factor associated with the development of CVE in patients with heart failure. For older patients with renal insufficiency, integrative or conventional therapy was not associated with the development of CVE. The integrative therapy of Chinese and Western medicine has a better long-term clinical outcome for patients.

## Figures and Tables

**Table 1:** Basic information of patients with heart failure in two groups.

	Integrative therapy *N* = 181	Conventional therapy *N* = 213	*P* ^*∗*^
Age (years)	63.98 ± 10.44	60.85 ± 11.25	**0.005**
Gender (male), cases (%)	107 (59.1)	141 (66.2)	0.174
Weight (kg)	71.55 ± 14.62	71.62 ± 14.59	0.964
BMI (kg·m^−2^)	25.48 ± 4.35	25.17 ± 3.94	0.500

Medication use (cases (%))			
Diuretics	150 (82.9)	172 (80.8)	0.587
ACEI/ARB	117 (64.6)	154 (72.3)	0.099
*β*-Blockers	143 (79.0)	181 (85.0)	0.107
MRA	129 (71.3)	135 (63.4)	0.129

Comorbidities (cases (%))			
Hypertension	122 (67.4)	139 (65.3)	0.747
Coronary artery disease	98 (54.1)	134 (62.9)	0.079
Arrhythmia	60 (33.1)	80 (37.6)	0.397
Heart valve disease	19 (10.5)	25 (11.7)	0.697
Diabetes mellitus	106 (58.6)	108 (50.7)	0.154
Renal insufficiency	76 (42.0)	45 (21.1)	**<0.001**

Smoking history (cases (%))	63 (34.8)	88 (41.3)	0.118
Hb (g·L^−1^)	127.48 ± 23.89	129.91 ± 25.19	0.330
Cr (*μ*mol·L^−1^)	92.30 (72.50, 130.30)	83.90 (69.30, 107.43)	**0.040**
TG (mmol·L^−1^)	1.67 ± 0.78	1.63 ± 1.04	0.681
LDL-C (mmol·L^−1^)	2.41 ± 0.80	2.42 ± 0.91	0.896
Hs-CRP (mg·L^−1^)	6.71 (2.13, 14.54)	3.00 (1.20, 11.61)	**0.007**
NT-proBNP (pg·mL^−1^)	2455.00 (625.00, 7400.50)	1520.00 (452.00, 3594.00)	**0.019**
Hcy (*μ*mol·L^−1^)	17.98 ± 8.50	17.64 ± 13.26	0.823
K^+^ (mmol·L^−1^)	4.06 ± 0.51	4.04 ± 0.48	0.681
Na^+^ (mmol·L^−1^)	139.69 ± 3.24	139.26 ± 3.12	0.203
Cl^−^ (mmol·L^−1^)	103.14 ± 8.07	103.67 ± 4.21	0.413
Mg^2+^ (mmol·L^−1^)	0.88 ± 0.11	0.89 ± 0.10	0.362

Echocardiography index			
LVEF (%)	47.45 ± 15.03	49.36 ± 14.09	0.215
LVMI (g·m^−2^)	122.20 ± 42.73	116.93 ± 35.26	0.239

In-hospital cardiac function class (cases (%))			**0.004**
I	0 (0)	0 (0)	
II	19 (10.5)	27 (12.7)	
III	73 (40.3)	116 (54.5)	
IV	89 (49.2)	70 (32.8)	
Hospital days (days)	9.76 ± 4.10	8.43 ± 4.77	**0.003**

^*∗*^Differential analysis of indicators between patients in the integrative therapy group and conventional therapy group.

**Table 2:** Survival table based on Kaplan–Meier method.

	Average survival time (months)	Median survival time (months)	*χ* ^2^	*P* ^*∗*^
All patients, *N* = 394	19.94 (18.40, 21.47)	17.00 (13.91, 20.09)	12.58	**<0.001**
Integrative therapy, *N* = 181	22.57 (20.50, 24.64)	21.00 (14.54, 27.46)		
Conventional therapy, *N* = 213	17.42 (15.12, 19.73)	14.00 (11.36, 16.63)		

^*∗*^Log rank method was used to test the difference of survival curves between the two groups of patients.

**Table 3:** Cox regression analysis of different “therapy modalities” and the occurrence of CVE in heart failure patients.

	Integrative therapy^*∗*^	Conventional therapy	*P*
Outcome events (cases (%))	90 (49.7)	113 (53.1)	0.545
Model I^1^	0.609 (0.458, 0.808)	1.00 (ref.)	**0.001**
Model II^2^	0.353 (0.218, 0.573)	1.00 (ref.)	**<0.001**
Model III^3^	0.322 (0.185, 0.561)	1.00 (ref.)	**<0.001**

^*∗*^Risk ratio (HR) and 95% confidence interval (95% CI): calculated by Cox proportional risk regression model, compared to the conventional therapy group. ^1^Model I was adjusted according to age and gender. ^2^Model II was adjusted for BMI, combined medications, past medical history, smoking history, comorbidities, and basal cardiac function class on the basis of model I. ^3^Model III was further adjusted for continuous variables, such as laboratory test results, LVEF, and LVMI.

**Table 4:** Subgroup analysis of treatment modalities and CVE occurrence in patients with heart failure.

	Total patients (cases)	Integrative therapy (cases)	HR (95% CI)^*∗*^	*P*
Age (years)				
≥65	203	103	0.551 (0.217, 1.400)	0.210
<65	191	78	0.259 (0.108, 0.621)	**0.002**

Baseline cardiac function class				
II	46	19	0.207 (0.001, 116.507)	0.626
III	189	73	0.296 (0.128, 0.686)	**0.005**
IV	159	89	0.138 (0.038, 0.497)	**0.002**

Renal insufficiency				
Yes	121	76	0.480 (0.222, 1.038)	0.062
No	273	105	0.307 (0.159, 0.592)	**<0.001**

Coronary artery disease				
Yes	232	98	0.304 (0.149, 0.619)	**0.001**
No	162	83	0.282 (0.096, 0.830)	**0.022**

^*∗*^Calculated by Cox regression model III, compared to the conventional therapy group.

**Table 5:** Predicted overall frequencies of heart function status of patients in both groups in 5 years by Markov model.

NYHA	1 year of therapy^*∗*^		3 years of therapy^*∗*^		5 years of therapy^*∗*^	
	Integrative therapy	Conventional therapy	Integrative therapy	Conventional therapy	Integrative therapy	Conventional therapy
I	7.2	3.0	13.0	4.6	13.7	4.6
II	24.4	20.4	6.9	4.0	2.3	0.9
III	21.2	19.4	5.3	3.7	1.6	0.8
IV	4.9	3.1	1.2	0.6	0.3	0.1
CVE	42.3	54.1	73.6	87.1	82.1	93.6
*P* ^*∗∗*^	<0.001		<0.001		<0.001	

^*∗*^Frequency of distribution by state (%). ^∗∗^Comparison of the differences in the distribution of cardiac function between the two groups.

**Table 6:** Frequencies of actual and predicted heart function distribution in patients in two groups after 1 year of treatment.

	NYHA I^*∗*^		NYHA II^*∗*^		NYHA III^*∗*^		NYHA IV^*∗*^		CVE^*∗*^	
	Prediction	Actuality	Prediction	Actuality	Prediction	Actuality	Prediction	Actuality	Prediction	Actuality
Integrative therapy^a^	7.2	6.8	24.4	26.0	21.2	18.6	4.9	3.4	42.3	45.2
Conventional therapy^b^	3.0	2.9	20.4	22.2	19.4	21.0	3.1	1.4	54.1	52.5

^*∗*^Frequency of distribution by state (%). ^a^Predicted and actual values of “cardiac function class” in the integrative therapy group, chi-square test for difference *P*=0.200. ^b^Predicted and actual values of “cardiac function class” in the conventional therapy group, chi-square test for difference *P*=0.089.

## Data Availability

Portions of the original data may be available, upon request, to a limited extent. However, other data cannot be provided due to the confidentiality of patients' personal information.

## Abbreviations

HF: Heart failure
ACEI: Angiotensin-converting enzyme inhibitors
ARB: Angiotensin-receptor blockade
MRA: Mineralocorticoid receptor antagonist
CVE: Cardiovascular events
LVEF: Left ventricular ejection fraction
Hb: Haemoglobin
Hs-CRP: Hypersensitive C-reactive protein
LDL-C: Low-density lipoprotein cholesterol
NT-proBNP: N-terminal pro-B type natriuretic peptide
LVIDd: Left ventricular end-diastolic diameter
PWTd: Left ventricular posterior wall thickness
SWTd: Septal thickness
LVMI: Left ventricular mass index
LVM: Left ventricular mass
BSA: Body surface area
ROC: Receiver operating characteristic curve
BMI: Body mass index.

## Supplementary Materials

Supplementary Materials Table S1: Basic information on all patients with heart failure. Table S2: Transformation table of variables. Table S3: Frequency and frequency matrix of change between states at 6 months for patients in the integrative therapy group. Table S4: Frequency and frequency matrix of change between states at 6 months for patients in the conventional therapy group. Figure S1: Flowchart of patient inclusion. Flow of all patients in the study from screening to inclusion.
