# Supplementary material for: Effect of Integrative Chinese and Western Medicine Therapy on Long-Term Clinical Outcomes in Patients with Heart Failure: A Real-World Study Including 394 Patients
Source: Evid Based Complement Alternat Med. 2022 Sep 13;2022:2001397. doi: 10.1155/2022/2001397 (PMC9489340; doi:10.1155/2022/2001397)
Supplement: Supplementary Materials — Table S1: Basic information on all patients with heart failure. Table S2: Transformation table of variables. Table S3: Frequency and frequency matrix of change between states at 6 months for patients in the integrative therapy group. Table S4: Frequency and frequency matrix of change between states at 6 months for patients in the conventional therapy group. Figure S1: Flowchart of patient inclusion. Flow of all patients in the study from screening to inclusion. [file 2001397.f1.zip › Supplementary materials Figure S1.docx]

2016-07 to 2021-07

Confirmed Heart Failure Admitted Patients

N=761

Age > 75 years

N=132

Patients Not First Hospitalized

N=53

Combined with severe liver and kidney abnormalities, severe hematopoietic system and endocrine system diseases

N=34

Loss to follow-up, or an unable to complete follow-up due to mental or other factors

N=43

Patients who met the inclusion criteria

N=481

Patients who cannot determine their treatment plan

N=30

Patients with too many missing test results

N=57

Patients included in the study

N=394

Based on retrievable treatment options

Conventional therapy

N=213

Integrative therapy

N=181

Using intravenous preparations of traditional Chinese medicine and proprietary Chinese medicines, and using new anti-heart failure drugs such as sakubatril valsartan and SGLT2 inhibitors

N=18
