# Supplementary material for: Effect of Integrative Chinese and Western Medicine Therapy on Long-Term Clinical Outcomes in Patients with Heart Failure: A Real-World Study Including 394 Patients
Source: Evid Based Complement Alternat Med. 2022 Sep 13;2022:2001397. doi: 10.1155/2022/2001397 (PMC9489340; doi:10.1155/2022/2001397)
Supplement: Supplementary Materials — Table S1: Basic information on all patients with heart failure. Table S2: Transformation table of variables. Table S3: Frequency and frequency matrix of change between states at 6 months for patients in the integrative therapy group. Table S4: Frequency and frequency matrix of change between states at 6 months for patients in the conventional therapy group. Figure S1: Flowchart of patient inclusion. Flow of all patients in the study from screening to inclusion. [file 2001397.f1.zip › Supplementary materials Tables S1-S4.docx]

**Table S1**

|  | Total patients N=394 |
| --- | --- |
| Age / years | 62.29±10.98 |
| Gender / Male, cases (%) | 248 (62.9) |
| Weight /Kg | 71.59±14.58 |
| BMI /Kg·m^-2^ | 25.31±4.12 |
| Medication use / cases (%) |  |
| Diuretics | 322 (81.7) |
| ACEI/ARB | 271 (68.8) |
| β-blockers | 324 (82.2) |
| MRA | 264 (67.0) |
| Comorbidities / cases (%) |  |
| Hypertension | 261 (66.8) |
| Coronary artery disease | 232 (58.9) |
| Arrhythmia | 140 (35.8) |
| Heart valve disease | 44 (11.2) |
| Diabetes mellitus | 214 (54.7) |
| Renal insufficiency | 121 (30.7) |
| Smoking history / cases(%) | 151 (38.3) |
| Hb /g·L^-1^ | 131.82±23.72 |
| Cr /μmol·L^-1^ | 78.30 (68.25,103.20) |
| TG /mmol·L^-1^ | 1.73±1.04 |
| LDL-C /mmol·L^-1^ | 2.41±0.83 |
| Hs-CRP /mg·L^-1^ | 3.01 (1.23,8.94) |
| NT-proBNP /pg·mL^-1^ | 1394.00 (400.50,4899.00) |
| Hcy /μmol·L^-1^ | 17.79±11.42 |
| K^+^ /mmol·L^-1^ | 4.06±0.45 |
| Na^+^ /mmol·L^-1^ | 139.60±2.75 |
| Cl^-^ /mmol·L^-1^ | 103.99±3.91 |
| Mg^2+^ /mmol·L^-1^ | 0.88±0.10 |
| Echocardiography Index |  |
| LVEF /% | 48.48±14.54 |
| LVMI /g·m^-2^ | 117.84±38.97 |
| In-hospital cardiac function class / cases(%) |  |
| Ⅰ | 0 (0) |
| Ⅱ | 46 (11.7) |
| Ⅲ | 189 (48.0) |
| Ⅳ | 159 (40.3) |
| Hospital days /days | 9.16±4.64 |

**Table S2**

|  | Continuous variables | Categorical variables |
| --- | --- | --- |
| BMI /Kg·m^-2^ | BMI＜18.5 | Low BMI |
|  | 18.5≤BMI≤24 | Medium BMI |
|  | BMI＞24 | High BMI |
| Hb /g·L^-1^ | Hb＜110 | Low Hb |
|  | Hb≥110 | Normal Hb |
| TG /mmol·L^-1^ | TG＞1.70 | High TG |
|  | TG≤1.70 | Normal TG |
| LDL-C /mmol·L^-1^ | LDL-C＞1.8 | High LDL-C |
|  | LDL-C≤1.8 | Normal LDL-C |
| Hcy /μmol·L^-1^ | Hcy＞15 | High Hcy |
|  | Hcy≤15 | Normal Hcy |
| Hs-CRP /mg·L^-1^ | Hs-CRP＞3 | High Hs-CRP |
|  | Hs-CRP≤3 | Normal Hs-CRP |
| Na^+^ /mmol·L^-1^ | Na^+^＜135 | Electrolyte disorder |
|  | Na^+^＞155 | Electrolyte disorder |
|  | 135≤Na^+^≤155 | Normal Na^+^ |
| K^+^ /mmol·L^-1^ | K ^+^＜3.5 | Electrolyte disorder |
|  | K ^+^＞5.5 | Electrolyte disorder |
|  | 3.5≤K^+^≤5.5 | Normal K ^+^ |
| Mg^2+^ /mmol·L^-1^ | Mg^2+^＜0.78 | Electrolyte disorder |
|  | Mg^2+^＞1.27 | Electrolyte disorder |
|  | 0.78≤Mg^2+^≤1.27 | Normal Mg^2+^ |
| Cl^-^ /mmol·L^-1^ | Cl^-^＜90 | Electrolyte disorder |
|  | Cl^-^＞110 | Electrolyte disorder |
|  | 90≤Cl^-^≤110 | Normal Cl^-^ |

**Table S3**

| Baseline cardiac function class | Total  cases | Cardiac function classification at 6 months of therapy**^*^** | | | | |
| --- | --- | --- | --- | --- | --- | --- |
|  |  | NYHA Ⅰ | NYHA Ⅱ | NYHA Ⅲ | NYHA Ⅳ | CVE |
| NYHA Ⅰ | 0 | 0 | 0 | 0 | 0 | 0 |
| NYHA Ⅱ | 67 | 7 (10.4) | 34 (50.8) | 12 (17.9) | 1 (1.5) | 13 (19.4) |
| NYHA Ⅲ | 94 | 0 | 24 (25.5) | 31 (33.0) | 12 (12.8) | 27 (28.7) |
| NYHA Ⅳ | 18 | 0 | 0 | 12 (66.7) | 1 (5.6) | 5 (27.7) |
| CVE | 0 | 0 | 0 | 0 | 0 | 0 |

**^*^** Frequency of transfer of baseline cardiac functional status to a new status at 6 months of treatment (this frequency as a proportion of the total number of cases corresponding to baseline cardiac functional status).

**Table S4**

| Baseline cardiac function class | Total  cases | Cardiac function classification at 6 months of therapy **^*^** | | | | |
| --- | --- | --- | --- | --- | --- | --- |
|  |  | NYHAⅠ | NYHAⅡ | NYHAⅢ | NYHAⅣ | CVE |
| NYHAⅠ | 0 | 0 | 0 | 0 | 0 | 0 |
| NYHAⅡ | 100 | 3 (3.0) | 48 (48.0) | 22 (22.0) | 0 | 27 (27.0) |
| NYHAⅢ | 96 | 1 (1.0) | 18 (18.7) | 38 (39.6) | 9 (9.4) | 30 (31.3) |
| NYHAⅣ | 17 | 0 | 0 | 3 (17.6) | 1 (5.9) | 13 (76.5) |
| CVE | 0 | 0 | 0 | 0 | 0 | 0 |

**^*^**Frequency of transfer of baseline cardiac functional status to a new status at 6 months of treatment (this frequency as a proportion of the total number of cases corresponding to baseline cardiac functional status).
